# Using Omics to Understand and Treat Pulmonary Vascular Disease

**DOI:** 10.3389/fmed.2018.00157

**Published:** 2018-05-24

**Authors:** Anna R. Hemnes

**Affiliations:** Division of Allergy, Pulmonary and Critical Care Medicine, Vanderbilt University Medical Center, Nashville, TN, United States

**Keywords:** pulmonary hypertension, omics-technologies, precision medicine, right ventricle, OMICS data

## Abstract

Pulmonary arterial hypertension (PAH) is a devastating disease for which there is no cure. Presently this condition is differentiated from other diseases of the pulmonary vasculature by a practitioner's history, physical examination, and clinical studies with clinical markers of disease severity primarily guiding therapeutic choices. New technologies such as next generation DNA sequencing, high throughput RNA sequencing, metabolomics and proteomics have greatly enhanced the amount of data that can be studied efficiently in patients with PAH and other rare diseases. There is emerging data on the use of these “Omics” for pulmonary vascular disease classification and diagnosis and also new work that suggests molecular markers, including Omics, may be used to more efficiently match patients to their own most effective therapies. This review focuses on the state of knowledge on molecular classification and treatment of PAH. Strengths and weaknesses of current Omic technologies are discussed and how these new technologies can be used in the future to improve diagnosis of pulmonary vascular disease, more effectively treat patients with existing and future drugs, and generate new understanding of disease pathogenesis and mechanisms underlying treatment success or failure. Bioinformatic methods to analyze the large volumes of data are developing rapidly, but still present major challenges to interpretation of potential Omic findings in pulmonary vascular disease, with low numbers of patients studied and a potentially high false discovery rate. With more experience, precise and established drug response definitions, this field with move forward and will likely be a major component of the clinical care of PH patients in the future.

## Introduction

Despite major advances in the last several decades, pulmonary vascular disease is a major source of morbidity and mortality. Primarily manifest as elevated pulmonary arterial pressure or pulmonary hypertension (PH), pulmonary vascular disease also includes pulmonary embolism and vascular malformations. PH has been subdivided in the current classification scheme into conditions that have a primary pulmonary vasculopathy, e.g., pulmonary arterial hypertension (PAH) and chronic thromboembolic PH (CTEPH) and conditions in which PH is considered to be a secondary phenomenon (1). For instance, left atrial hypertension raises pulmonary venous pressure and subsequently pulmonary arterial pressure, but if the left atrial pressure were removed, the PH would regress, theoretically. Similarly, parenchymal lung disease such as chronic obstructive pulmonary disease is associated with PH occasionally, but pathologic specimens do not uniformly demonstrate pulmonary arterial vasculopathy. Many of these conditions predispose to right ventricular failure and ultimately result in death, despite new treatments, making improved diagnostic and therapeutic options potentially highly clinically impactful.

Some forms of PH, e.g., PAH and chronic thromboembolic pulmonary hypertension (CTEPH), have specific medical or surgical therapies (2, 3), however the majority of PH and pulmonary vascular disease in general have no therapy aside from supportive care. Further complicating this group of diseases is the lack of specific diagnostic material. Lung biopsy is generally considered high risk in patients with PH or other forms of pulmonary vascular disease (4, 5), making diagnosis reliant on clinical factors, radiographic findings and invasive hemodynamics. This leaves clinicians with a major challenge of potentially missing a treatable condition such as PAH or CTEPH when they diagnose other forms of PH. Alternatively, PAH-directed therapy has been shown to potentially harm patients with non-PAH PH (6, 7), thus a practice pattern of simply treating all PH as PAH is potentially dangerous. In short, the field is in need of improved diagnostic modalities and more efficient mechanisms through which to identify patients who respond to a specific treatment.

Recent advances in biomedical research and bioinformatics approaches are now available to apply to pulmonary vascular disease and hold tremendous promise to improve diagnostic accuracy and improve treatments of all forms of pulmonary vascular disease. The term “Omics,” hereafter referred to Omics, refers to the field of study generally ending in “-omics” such as genomics, proteomics, and transcriptomics. Broadly speaking, Omics suggests study of the entire field of proteins, metabolites or genes that may contribute to a disease state or phenomenon. Although early work in Omics included studies of genomics, transcriptomics, metabolomics, and proteomics, more recent work has added other fields such as radiology (radiomics) and cell biology (cell biomics). The use of transcriptomics and genomics in pulmonary vascular disease is most established, but recent work has drawn attention to how other Omics may be used to understand and optimally treat pulmonary vascular disease. This review will focus on these two key areas: First, use of Omics to classify PH and, second, use of Omics to optimize therapy in PAH and PH more broadly.

## Omics to classify PH

Originally developed in the second World Symposium on Pulmonary Hypertension in 1998, the classification of PH attempts to categorize patients by similar clinical and hemodynamic patters that correlate with specific pathologic findings and response to therapies (8). Although modifications have been made, the classification of PH continues to rely on clinical metrics (1). One notable exception to this is heritable PAH in which genetic mutations such as those in bone morphogenetic protein receptor type 2 (BMPR2) and other mutations are well-described (9–13) and may be used to assist in classification of patients.

Although the classification is generally quite useful in predicting response to PAH-directed therapies, many patients have features of multiple forms of PH, so-called “mixed” or “combined” PH. An example of a patient with combined group 1 and 2 PH is the 75 year old woman, an aunt in the blood line of a patient with heritable PAH who has normal lungs by chest imaging and spirometry, no evidence of chronic thromboembolic PH by ventilation perfusion lung scan, and a right heart catheterization demonstrating a pulmonary arterial wedge pressure of 20 mmHg and pulmonary arterial pressure of 95/50 mmHg with a normal cardiac output. Her elevated pulmonary arterial wedge pressure suggests group 2 PH, however she has a wide diastolic pressure gradient, suggesting underling pulmonary vasculopathy. Further, she likely carries a BMPR2 mutation, making PAH possible. After the 2012 World Symposium on PH, this patient would be called combined pre- and post-capillary PH (Cpc-PH) (14) and her case demonstrates a common clinical scenario of a patient with multiple potential etiologies of PH. As lung biopsies are not routinely obtained due to risk of bleeding and increased surgical risk in PH patients (3, 4), precise classification of many patients' pulmonary vascular disease may not be possible. Further, since most medical therapy is reserved for patients with exclusively PAH (2, 3, 15), patients with indicators they may have other forms of PH in addition to PAH may not be recommended for life-saving therapies. Thus, there are significant limitations to the current clinical classification of PH.

Although specific gene sequencing for mutations in BMPR2, ALK1, and other genes can, in the right clinical context, confirm a diagnosis of heritable PAH, these are specific, targeted tests and only applicable to small number of even patients with PAH, and a minute fraction of PH in general (9, 16, 17). Recently there has been interest in using Omic technologies to enhance the current classification of PH, thus moving beyond tests of single gene mutations. Early work in this field has used multiple Omic technologies to understand primarily how PAH patients are different from healthy controls. Given that PAH can be challenging to diagnose and is a relatively heterogeneous group of diseases including congenital heart disease and multiple different connective tissue diseases, some of the earliest Omic work focused on heritable PAH, in which the diagnosis is firm and the disease etiology less heterogeneous. West and colleagues used microarray analysis on cultured peripheral blood lymphocytes to define RNA transcript patterns in patients with heritable PAH compared with controls (18). Cultured lymphocytes offer the advantage of several weeks of *ex vivo* culture, thereby removing environmental influences on the cells. These data demonstrated alterations in estrogen metabolism, actin organization, growth, and apoptosis signaling with significant differences between heritable PAH and control patients. These early studies used a very well-defined disease phenotype to compare to controls and served as a prototype for future Omic analysis, focusing on tight phenotype and pathway or gene ontology analysis.

More recently, Rhodes and colleagues performed a comprehensive study of 1,416 plasma metabolites using ultraperformance liquid chromatography mass spectrometry in patients with PAH and healthy controls (19). They included only idiopathic and heritable PAH patients, again ensuring a relatively homogenous population and compared with both healthy and disease controls. Using an unbiased approach to analysis, they identified metabolites that discern PAH from controls and, after correcting for various factors, found 20 metabolites that distinguish PAH from healthy and disease controls. A network analysis of these metabolites highlights alterations in amino acid, nucleoside and glucose and lipid metabolism. They further developed a discriminant score using seven metabolites to separate PAH from healthy and disease controls. Taken together, these data demonstrate that metabolomics can be used to define detectable, metabolic differences between PAH and control patients and perhaps point to pathways key to development or maintenance of PAH.

Our own group has used genomics to understand whether patients with Cpc-PH have genetic variant patterns that are more similar to PAH than to group 2 PH with isolated post-capillary PH (Ipc-PH). Assad and colleagues first defined demographics in the three groups of patients and showed the Cpc-PH is distinct and characterized by younger age than Ipc-PH patients with more severe pulmonary hemodynamics (20). Using a DNA biorepository with pre-existing data on single nucleotide variants linked to de-identified clinical data, distinct gene variant patterns in Cpc-PH were identified that were more similar to PAH than to Ipc-PH patients and were in pathways known to be of relevance to PAH such as extracellular matrix and immune function. These data show that there may be genetic variant patterns that could be used to both understand etiology of pulmonary vascular disease, but also to define phenotypes and endophenotypes of pulmonary vascular disease.

Other groups have used similar methodologies to explore expression patterns in the lung, clearly a highly relevant tissue to pulmonary vascular disease. Geraci and colleagues demonstrated different expression patterns in PAH compared to controls in lung tissue (21). Others have used Omics in tissue and peripheral blood to study differences in RNA expression patterns in scleroderma-associated PAH (22, 23). Taken together, these data show the broad applicability of RNA expression pattern studies to pulmonary vascular disease, both in the peripheral blood and the affected tissues. Unfortunately, due to low numbers of patients undergoing lung transplantation or autopsy, studies of tissues are limited in number, thereby potentially limiting data generated and increasing false discovery rate. Nonetheless, when available, tissue can provide key insights into disease pathology and also critical confirmatory information of peripheral blood findings.

There is much enthusiasm that Omics will 1 day either augment or replace our current clinical classification of PH. With large numbers of patient enrollees, new molecular classifications discovered using Omics, may allow for more precise diagnoses, similar to progress made in cancer diagnostics. With homogenous diagnostic categories, discovery of molecular etiologies, identification of new therapeutics and more targeted clinical trials will all be possible (24). The National Institutes of Health/National Heart, Lung and Blood Institute (NHLBI) has funded a multi-center United States cohort study to use Omics, including genomics, transcriptomics, proteomics, and metabolomics, among other Omics, to advance understanding and classification of PH patients and is presently enrolling (NCT02980887) (25). Studies such as these potentially will change the classification and diagnosis of PH type to one that incorporates measurement of blood-based metrics into clinical parameters to define disease etiology and treatment recommendations.

## Omics to improve therapy in PH

PAH is a specific form of PH in which the distal pulmonary arterioles develop occlusive lesions, termed plexiform lesions, as well as other changes resulting in elevations in pulmonary vascular resistance generally with right heart failure and death within 3–5 years if untreated. While major progress has been made in therapy of PAH in the last several decades, it remains a highly mortal disease, with a recent publication showing that despite modern therapy, 40% of newly diagnosed patients die within 5 years (26), survival similar to early stage lung cancer. There are 10 FDA-approved therapies in three broad classes, however, clinicians have a very limited capacity to determine which patients are likely to respond to which drug class. This leads to costly prescription of multiple classes of medications, greater exposure to side effects of medications and greater burden of therapy, without clear therapeutic benefit. While the combination of tadalafil and ambrisentan has been shown to improve time to clinical worsening (27), there is very limited data on the efficacy of other combinations of medications. Additionally, PAH therapy is expensive, with annual costs ranging from $20,000 to $1,000,000 per patient. Use of costly and potentially ineffective drugs is untenable for healthcare systems. Thus, there is a pressing need to develop novel treatment strategies in PAH, ideally using drugs that are presently FDA-approved in the short term, and applying these strategies to future pivotal trials of drugs in all forms of PH.

Omics technologies have been used to improve patient care through identification of markers or patterns of markers that indicate positive responses to specific therapies, often drugs. While occasionally the same marker that defines a disease state can be used to predict treatment responses, e.g., the presence of cystic fibrosis with G551D mutation responds to treatment with ivacaftor (28), it is possible and perhaps likely that markers of drug responsiveness are different from those of disease diagnosis. Here we focus on Omic predictors of responses to PAH therapies, as there are presently no data for other forms of PH.

Some forms of PAH may provide clues as to how we can more effectively and efficiently treat PH. It has long been recognized that about 5–10% of patients with PAH have an acute pulmonary vasodilatory response when confronted with vasodilator therapy with normalization or near-normalization of pulmonary arterial pressures and preservation of cardiac output (29). Patients meeting these criteria can be treated safely with calcium channel blocker medications with substantially improved mortality and a much cheaper drug treatment cost (30). The identification of acute vasodilator response as a predictor of long term response to calcium channel blocker therapy was a key development in the therapy of PAH as it (1) was the first foray into precision medicine in the field of pulmonary vascular disease and (2) defined a phenotypically homogenous group of drug responders that later could be studied to understand molecular etiology and markers of drug responsiveness in PAH.

Years after the discovery of this distinct phenotype, Omic technologies had advanced such that their use to find predictors of drug responsiveness now seemed plausible. Our group used transcriptomics to determine if the clinical response to calcium channel blocker therapies could be predicted using molecular markers (32). A major limitation to studying this condition is its extreme rarity making study of untreated patients infeasible. We overcame this limitation using cultured lymphocytes as above. Because the lymphocytes were cultured *ex vivo* for weeks, potential confounding by exposure to calcium channel blockers was minimized. We used microarray to measure mRNA expression levels and found that there were distinct patterns of RNA expression in the vasodilator-responsive PAH patients compared to those that were not. Since our primary goal was to define a simple RNA expression pattern to predict vasodilator responsive PAH, we confirmed the most differentially expressed genes using whole blood RNA. After doing this, we developed an algorithm of RNA expression patterns that predicted this drug-responsive PAH phenotype and validated it in an external cohort. The strengths of this approach are that the RNAs were selected agnostic of their biologic plausibility, allowing the selection of the strongest predictors and the use of cultured lymphocytes to remove environmental influences on RNA patterns. These data suggested that in a cohort of tightly phenotyped drug responders, with a clear definition of drug response, Omic predictors might potentially be used to predict drug responses.

The most appealing Omic field for predicting drug responses is genomics. Generally not impacted by external forces or intercurrent illness, DNA is also more stable facilitating transfer to different facilities and readily available through blood draws or buccal mucosa swab. In addition, DNA would not be expected to change if a patient is already started on PAH-directed therapy, whereas metabolomics, proteomics, and transcriptomics may be altered by the therapy itself. Given these appealing qualities, there have been a few recent attempts to predict drug responses using genomics. First, Benza and colleagues used targeted DNA sequencing to study gene variants in the endothelin signaling pathway (33). They identified several variants that predict clinical outcomes with endothelin receptor antagonist therapy in PAH. The authors approach was limited to only genes known to be in the endothelin pathway and it is possible that with a discovery-based approach they would have identified even stronger predictors of endothelin receptor antagonist therapy outcomes.

Our group used whole exome sequencing in PAH patients with and without acute vasodilatory response. Again using this distinct endophenotype as a proof of the concept that genomics can be used to predict drug responses. We did not identify one single gene or gene variant that predicts calcium channel blocker responses, however we did find over 1,500 DNA variants unique to PAH patients. Next generation DNA sequencing is well known to identify hundreds of variants of undetermined significance, a limitation we attempted to avoid using pathway analysis of identified variants, comparing patients with acute response to vasodilators and those without. After filtering out gene variants associated with the presence of idiopathic pulmonary fibrosis and those not predicted to be deleterious using online resources, we studied the variants based on their frequency and found that there were generally more gene variants in patients with vasodilator-responsive PAH and also these genes were enriched in pathways associated with smooth muscle cell contraction. In the case of vasodilator-responsive PAH, we learned that there likely is not a single gene that differentiates PAH from control, but more likely a pattern of gene variants. Perhaps with greater numbers, these data can be refined and a gene variant risk score for PAH may be developed. Presently, these drug Omic methodologies are not appropriate for clinical care, though hopefully their use is not far away.

**Figure 1:**
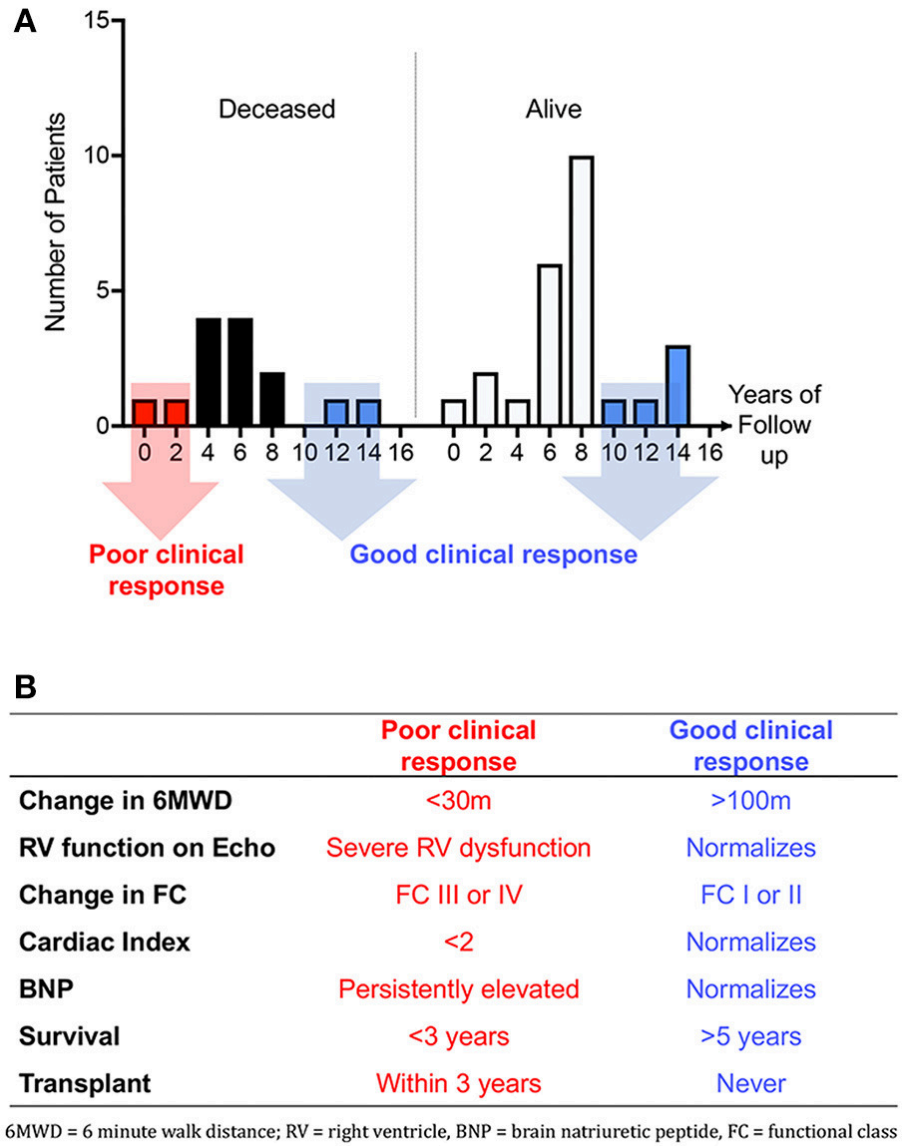
**(A)** Histograms of survival in ongoing survivors (*n* = 25) vs. deceased patients (*n* = 14). Y axis depicts years since initiation of IV epoprostenol. Patients with good response may be considered those still alive after 8 years of drug or those who are deceased but after 8 years of IV epoprostenol therapy. Eight years is the median survival in our cohort (31). **(B)** Table showing proposed single or combined metrics of response to parenteral prostacyclin therapy.

**Figure 2:**
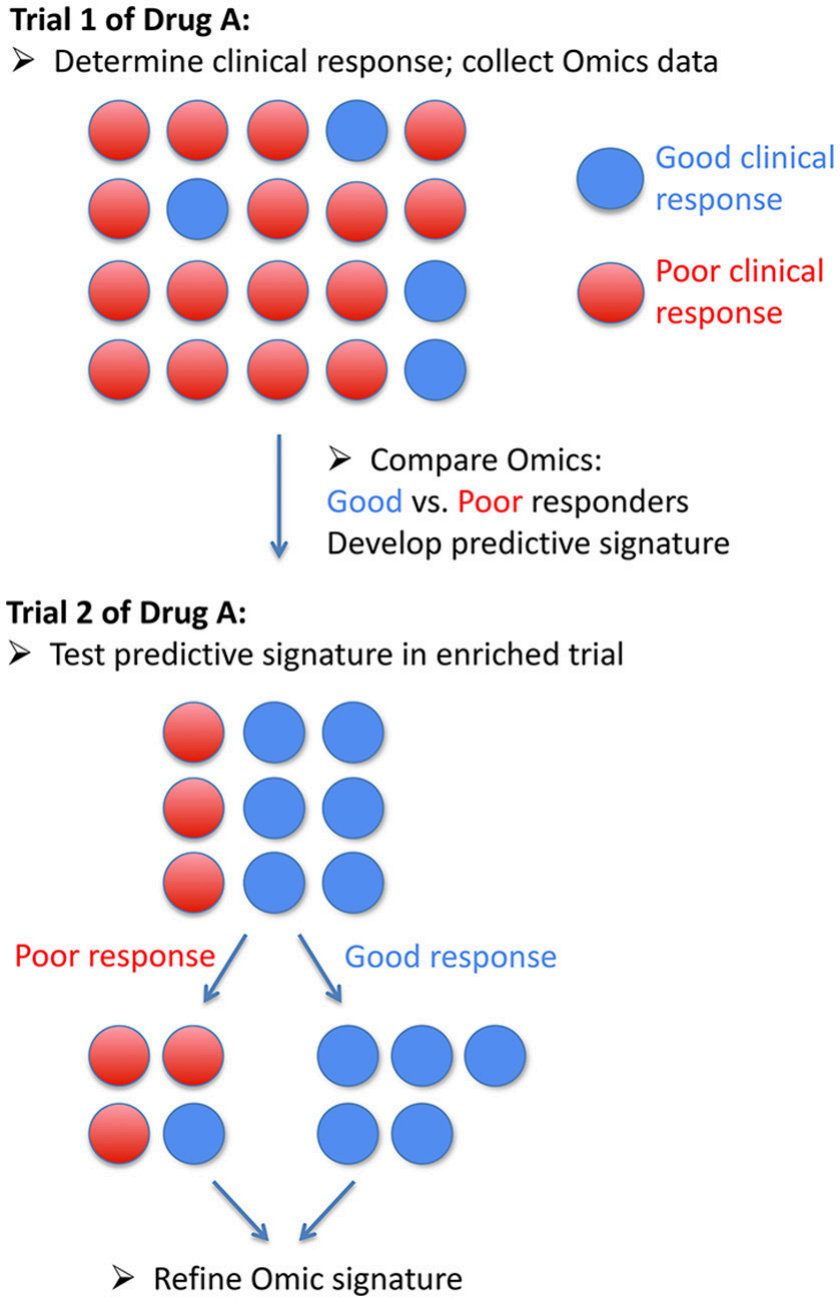
Schematic of how Omic data could be used in a precision medicine trial of a drug in pulmonary arterial hypertension.

Future areas in which Omics may be useful in PAH therapeutics include development of genetic or other markers of predictors of response to endothelin receptor antagonists, phosphodiesterase type 5 inhibitors, riociguat, and prostacyclin pathway therapeutics. Prostacyclins may be particularly appealing for this sort of analysis as excellent clinical response are well described, and endpoints such as death and transplant are unfortunately relatively common, making numbers of enrollees required to generate useful data relatively low (34). Examples of how groups may be defined, either singly or in combination, are shown in Figure 1, including a demonstration of how survival (Figure 1A) or clinical metrics (Figure 1B) could be used to define good or poor clinical response. Figure 2 outlines a schema of how Omics could be used to refine future trials in pulmonary hypertension medications, both existing and future.

## Challenges in the use of omics in pulmonary vascular disease

In order to move this field forward, we need a commonly agreed upon definition of “good response” to drugs to allow segregation of groups. A key feature that made analysis of RNA expression patterns in PAH patients with and without acute vasodilator responses possible was the distinct acute drug responses and long term survival data. This clear endophenotype could be exploited on a molecular level. However, most clinical drug responses are less well-established, with perhaps the exception of the occasional exceptional prostacyclin response. Moreover, use of a single drug to treat PAH is becoming less common, making identification of molecular predictors of response to a single drug less likely. Further, while Omics may select one or several biomarkers of prognosis in PAH, they must be carefully tested in their predictive value against traditional, highly predictive clinical models of survival in PAH such as the REVEAL risk score (26, 35, 36).

Further, we need to refine our pipeline for analysis of Omics data. Each methodology generates at a minimum, hundreds and, more commonly, thousands of data points that have to be filtered and prioritized. The use of biologic plausibility may lose strongly predictive Omic metrics and alternatively a simple “strength of expression” analysis may miss biologically important molecules with low level expression. Finally, integration of data across Omic platforms is presently challenging and required advanced computational skills with different analytic techniques. This has the potential to bias the final data and its interpretation. Thus different methods for analyzing data may yield very different results, each with its own strengths and weaknesses. Recent developments such as predictive modeling and machine learning, reviewed here (37) and here (38), may be a powerful way forward to integrate and understand these large datasets. Detailed discussion of these methodologies and their applications are outside the scope of this manuscript.

## Conclusion

In conclusion, Omic technologies hold tremendous promise to improve the diagnosis and treatment of PH. There are major limitations to their present use, however, including potentially high false discovery rate with low numbers of patients, imprecise phenotypes and bioinformatics challenges. With more experience, precise and established drug response definitions, this field with move forward and will likely be a major component of the clinical care of PH patients in the future.

## Author contributions

The author confirms being the sole contributor of this work and approved it for publication.

### Conflict of interest statement

The author declares that the research was conducted in the absence of any commercial or financial relationships that could be construed as a potential conflict of interest.
